# The histone demethylase KDM4B regulates peritoneal seeding of ovarian cancer

**DOI:** 10.1038/onc.2016.412

**Published:** 2016-11-21

**Authors:** C Wilson, L Qiu, Y Hong, T Karnik, G Tadros, B Mau, T Ma, Y Mu, J New, R J Louie, S Gunewardena, A K Godwin, O W Tawfik, J Chien, K F Roby, A J Krieg

**Affiliations:** 1 Department of Obstetrics and Gynecology, University of Kansas Medical Center, Kansas City, KS, USA; 2 Department of Pathology and Laboratory Medicine, University of Kansas Medical Center, Kansas City, KS, USA; 3 Medical Scholars Program, University of Kansas Medical Center, Kansas City, KS, USA; 4 Department of Radiation Oncology, University of California, San Francisco, San Francisco, CA, USA; 5 Department of Molecular and Integrative Physiology, University of Kansas Medical Center, Kansas City, KS, USA; 6 Department of Cancer Biology, University of Kansas Medical Center, Kansas City, KS, USA; 7 Department of Anatomy and Cell Biology, University of Kansas Medical Center, Kansas City, KS, USA

## Abstract

Epithelial ovarian cancer (EOC) has poor prognosis and rapid recurrence because of widespread dissemination of peritoneal metastases at diagnosis. Multiple pathways contribute to the aggressiveness of ovarian cancer, including hypoxic signaling mechanisms. In this study, we have determined that the hypoxia-inducible histone demethylase KDM4B is expressed in ∼60% of EOC tumors assayed, including primary and matched metastatic tumors. Expression of KDM4B in tumors is positively correlated with expression of the tumor hypoxia marker CA-IX, and is robustly induced in EOC cell lines exposed to hypoxia. KDM4B regulates expression of metastatic genes and pathways, and loss of KDM4B increases H3K9 trimethylation at the promoters of target genes like *LOXL2*, *LCN2* and *PDGFB*. Suppressing KDM4B inhibits ovarian cancer cell invasion, migration and spheroid formation *in vitro*. KDM4B also regulates seeding and growth of peritoneal tumors *in vivo*, where its expression corresponds to hypoxic regions. This is the first demonstration that a Jumonji-domain histone demethylase regulates cellular processes required for peritoneal dissemination of cancer cells, one of the predominant factors affecting prognosis of EOC. The pathways regulated by KDM4B may present novel opportunities to develop combinatorial therapies to improve existing therapies for EOC patients.

## Introduction

Epithelial ovarian cancer (EOC) is the most deadly of gynecologic cancers, and is the fifth leading cause of cancer mortality in women.^1^ EOC is a heterogeneous disease, generally stratified by the aggressiveness of growth, with slower growing type I tumors having better prognosis than more rapidly growing type II tumors.^2^ The majority of type II EOC patients are diagnosed at an advanced International Federation of Gynecology and Obstetrics stage (FIGO stage III or IV), with widespread dissemination of metastatic tumors throughout the peritoneal cavity.^2, 3, 4^ Of the four general pathological subtypes that comprise EOC (serous, endometrioid, mucinous and clear cell), the majority of type II EOC are high-grade serous adenocarcinoma (HGSA), representing 70% of the total EOC cases.^2, 4^ Front-line therapy for HGSA consists of surgical debulking followed by treatment with platinating agents and taxol derivatives.^4^ The initial response to therapy is generally effective; however, recurrent chemo-resistant tumors reseed the peritoneal cavity within 2 to 5 years, causing mortality. Rapid re-establishment of tumor growth suggests that the stresses imposed by the metastatic process, surgery and chemotherapy drive adaptations that facilitate EOC progression.

The hypoxic tumor microenvironment is a potent contributor to malignancy in multiple cancer types, including EOC.^5, 6, 7^ The hypoxia-inducible factors (HIFs) are primary regulators of the hypoxic response, inducing transcription of genes that promote homeostasis and tumor progression via metabolic adaptation, self-renewal, angiogenesis and metastasis.^5^ HIF-1α also induces expression of several Jumonji-domain histone demethylases (JmjC-KDMs), including *KDM4B* (lysine-specific histone demethylase 4B, also called JMJD2B), establishing a compelling link between the hypoxic tumor microenvironment and epigenetic remodeling to support tumorigenesis.^8, 9, 10, 11^ KDM4B is thought to regulate gene expression by demethylating tri- and di-methylated histone H3 at lysine 9 (H3K9me3/me2) and lysine 36 (H3K36me3/me2).^12^ Despite the importance of KDM4B to general tumor growth, its contribution to EOC progression has not been investigated. As HIF-1α and hypoxic signaling contribute to EOC,^7^ KDM4B may also influence EOC progression. Using immunohistochemical analysis of patient samples and *in vitro* and *in vivo* functional analyses of ovarian cancer cell lines, we have determined that KDM4B is abundantly expressed in EOC tumors, and contributes to multiple pathways required for peritoneal seeding. Thus, KDM4B represents a novel link between the hypoxic tumor microenvironment and EOC, mobilizing mechanisms that facilitate the establishment of peritoneal tumors.

## Results

### KDM4B is abundantly expressed in hypoxic high-grade ovarian serous adenocarcinoma tumors and OVCAR cell lines

**Figure 1:**
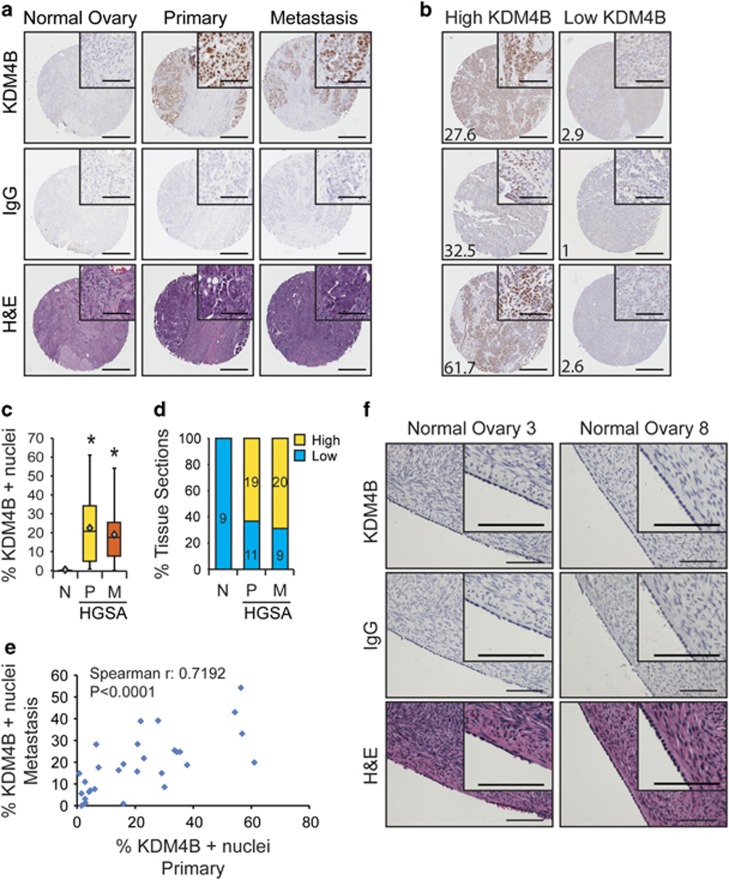
KDM4B is robustly expressed in ovarian cancer tissue and ovarian cancer cell lines compared with normal ovarian surface epithelial cells. (**a**) Representative immunohistochemical (IHC) detection of KDM4B (top row; brown, DAB; blue, hematoxylin) in tumor microarray sections from normal ovary and high-grade ovarian serous adenocarcinoma biopsies with matched primary and metastatic tumor sections. IgG (middle) and H&E (hematoxylin and eosin, bottom) serve as controls for nonspecific DAB staining and tissue structure controls, respectively. (**b**) Representative IHC images of tumor sections with high (left) and low (right) KDM4B. For each image, the percentage of cancer cell nuclei staining positive for KDM4B is denoted in the lower left corner. For (**a**) and (**b**), scale bar for entire section, 100 μm; scale bar for inset, 50 μm. (**c**) Box-and-whisker plot of KDM4B nuclear staining in normal ovary (N, *n*=9), primary HGSA tumors (P, *n*=30) and metastases (M, *n*=29). Diamond, mean; significance of staining differences was determined using Kruskal–Wallis, with Mann–Whitney *U*-test (*P<0.001). (**d**) Percentage of total patient samples in each category (normal, primary and metastasis) staining for high or low KDM4B (high >10% nuclei staining positive for KDM4B, low <10%). (**e**) Correlation between KDM4B staining in metastases (*n*=29) compared with matched primary tumors (*n*=29). Significance determined by Spearman's correlation. (**f**) KDM4B expression in representative normal ovarian surface epithelium. Scale bar, 100 μm.

Hypoxic regulation of KDM4B is a common mechanism in many different cancer cell types,^9, 10, 11, 13^ suggesting that KDM4B would also be robustly expressed in EOC. Expression of KDM4B was measured in ovarian cancer tissue microarrays (TMAs; see Materials and methods and Supplementary Tables S1 and S2). Nuclear KDM4B staining was greater in tumor epithelium of HGSA tumors compared with normal ovarian sections (Figure 1a, quantified in Figure 1c). Over 60% of primary and metastatic tumors displayed ‘high' expression of KDM4B (Figure 1b, quantified in Figure 1d, see Materials and methods). Nuclear expression of KDM4B in metastatic tumors positively correlated with KDM4B expression in the corresponding primary tumors (Figure 1e, Spearman's *r*=0.7192, *P*<0.0001). Independent measurements using sections from nine normal ovaries showed weak KDM4B staining in normal ovarian surface epithelium (Figure 1f), one potential source tissue for EOC.^4^

**Figure 2:**
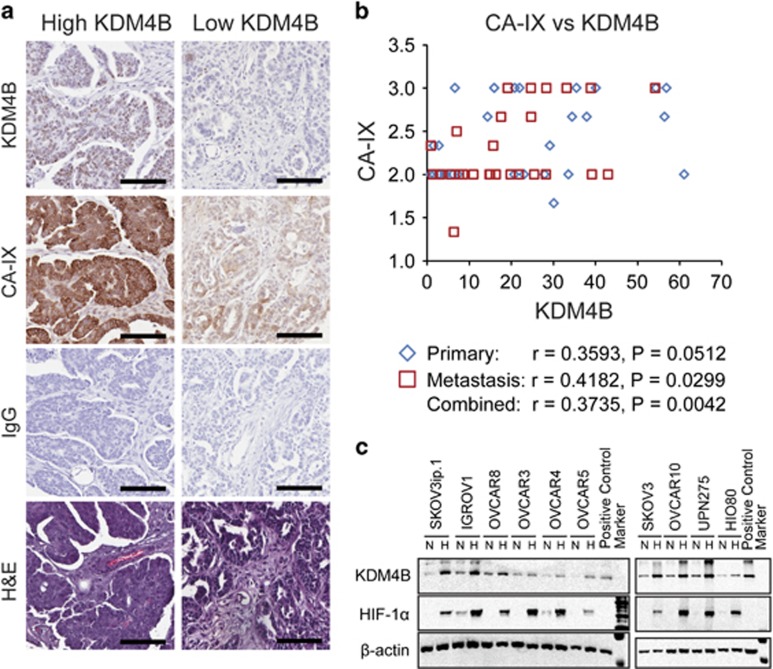
KDM4B is expressed in hypoxic ovarian cancer tumors and EOC cell lines. (**a**) Representative images of TMA sections demonstrating association between elevated KDM4B staining with elevated CA-IX staining. IgG and hematoxylin and eosin (H&E) serve as negative and tissue structure controls, respectively. Scale bar, 50 μm. (**b**) Scatter plot of hypoxia (CA-IX staining) vs KDM4B staining in TMA sections. Blue diamonds, primary tumors (*n*=30); red squares, metastatic tumors (*n*=29). Statistical significance determined using Spearman's correlation. *P-*value of <0.05 is considered significant. (**c**) Immunoblot detection of KDM4B and HIF-1α in a panel of ovarian cancer cell lines in normoxia (N, 21% oxygen) and in hypoxia (H, 0.5% oxygen). HIO-80 cells serve as nontransformed ovarian surface epithelium (OSE) control. HIF-1α indicates cellular hypoxia, β-actin serves as loading control.

The expression of the tumor hypoxia marker carbonic anhydrase 9 (CA-IX) was also scored in the TMAs (Figures 2a and b and Supplementary Table S2). Average intensity of CA-IX staining for triplicate tumor sections in the HGSA tumors (range 0–3) was positively correlated to the percentage of KDM4B-positive tumor nuclei (Figure 2b). When the primary and metastatic tumors were analyzed separately, this correlation extended to each group, although significance was achieved only in the metastases (*P*=0.03). In addition, KDM4B was robustly expressed in multiple cell lines representing a broad range of EOC genotypes, particularly in response to hypoxia (0.5% oxygen, Figure 2c). There was weak hypoxic induction of KDM4B in the immortalized ovarian surface epithelium cell line HIO80 (Figure 2c). KDM4B is expressed in hypoxic EOC cells, and may contribute to progression of HGSA.

### KDM4B differentially regulates gene expression in atmospheric and hypoxic growth conditions

**Figure 3:**
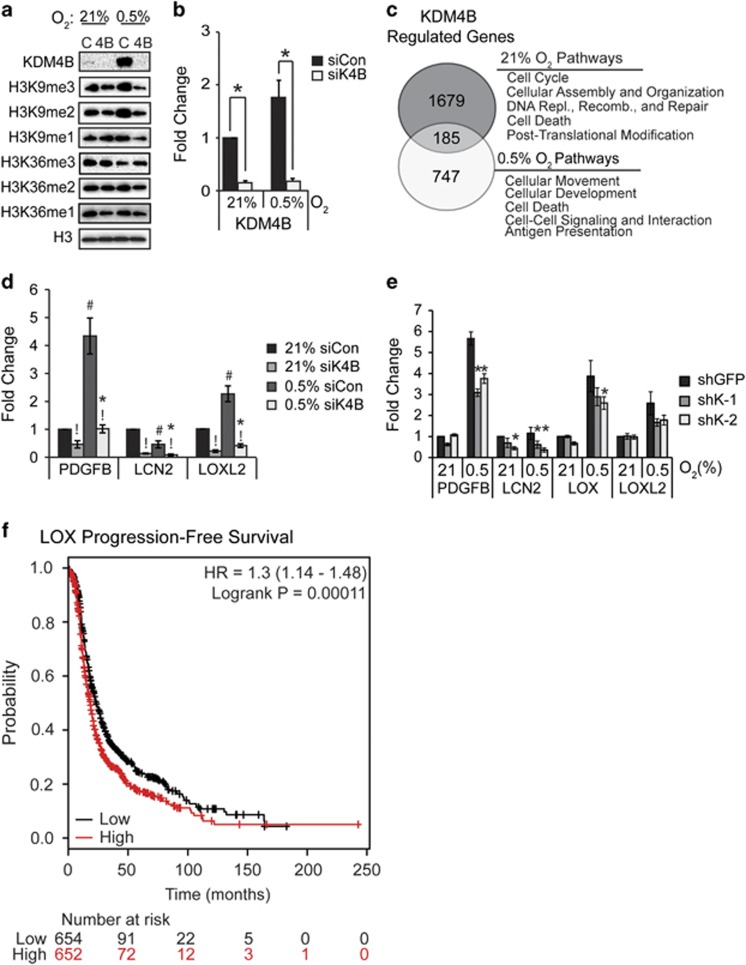
KDM4B regulates different pathways in normoxia and hypoxia. (**a**) Immunoblotting of KDM4B expression, H3K9me3, H3K9me2, H3K9me1, H3K36me3, H3K36me2 and H3K36me1 in SKOV3ip.1 cells transfected with small interfering RNA (siRNA) to KDM4B (4B) or control siRNA (C). Histone H3 serves as control for protein loading. (**b**) Quantitative real-time PCR (RT–PCR) of KDM4B expression in SKOV3ip.1 cells transfected with siRNA to KDM4B (siK4B) or control siRNA (siCon) and exposed to 21 or 0.5% oxygen for 16 h. (**c**) Venn diagram showing the overlap between genes downregulated greater than 1.4-fold by siK4B in normoxia (top circle) and hypoxia (bottom circle). (**d**) Quantitative RT–PCR validation of selected metastasis-associated genes regulated by KDM4B. Data in (**b**) and (**d**) represent mean fold change±s.e.m. normalized to 18S rRNA, calculated relative to siCon in 21% O_2_ (black bars). Results were averaged from three independent experiments, measured in triplicate. Significance of differences was calculated using two-tailed paired Student's *t*-test (^!^*P*<0.05 for siK4B compared with siCon; ^#^*P*<0.05 for hypoxia compared with normoxia) or two-way analysis of variance (ANOVA) (*P<0.05 for interactions between oxygen conditions and KDM4B expression). (**e**) Quantitative RT–PCR measurement of *PDGFB*, *LCN2* and *LOX* in SKOV3ip.1 cells expressing shRNA to KDM4B (shK-1, dark gray and shK-2, light gray) cultured in the indicated oxygen tensions. Data in (**e**) represent mean fold change±s.e.m., normalized to 18S rRNA and shGFP control at 21% oxygen (*n*=5 measured in triplicate). *P<0.05, determined by two-tailed paired Student's *t*-test, relative to shGFP control at the respective oxygen tension. (**f**) Kaplan–Meier progression-free survival plot of LOX expression in all stages of serous epithelial ovarian cancer from data set curated by Gyorffy *et al.*^20^ (*P*<0.05).

In order to identify genes regulated by KDM4B in EOC, SKOV3ip.1 cells were transiently transfected with small interfering RNA to KDM4B (siK4B) or an irrelevant control (siCon), achieving robust knockdown in both normoxia (21% oxygen) and hypoxia (0.5% oxygen; Figures 3a and b). SKOV3ip.1 cells form peritoneal xenograft tumors in a manner consistent with human EOC, facilitating translation of gene expression data to functional analysis.^14, 15^ Consistent with prior reports,^9^ loss of KDM4B did not increase bulk H3K9 trimethylation (H3K9me3, antibody specificity demonstrated in Supplementary Figure S1) or dimethylation (H3K9me2) in SKOV3ip.1 or OVCAR8 cell lines (Figure 3a and Supplementary Figure S2A, respectively). Similar results were observed for bulk H3K36 trimethylation and dimethylation (Figure 3a and Supplementary Figure S2A). These results suggest that KDM4B regulates gene expression through specific mechanisms rather than global shifts in chromatin modification.^9^ Genes dependent on KDM4B in 21 or 0.5% oxygen were identified by expression microarray analysis (see Materials and methods). KDM4B positively regulated expression of 1864 gene probes in normoxia, and 932 gene probes in hypoxia, with an overlap of 185 genes regulated in both conditions (Figure 3c). The pathways regulated by KDM4B in each oxygen condition were distinctly different (Supplementary Table S3). In normoxia, KDM4B predominantly regulated genes involved in cancer, cell cycle (including DNA replication, recombination and repair) and cell death pathways. In hypoxia, KDM4B regulated genes associated with inflammatory response, cellular development and cellular movement, implying a possible role in metastasis (see Supplementary Tables S4–S6 for complete lists of KDM4B-dependent and hypoxia-inducible gene expression). Several genes were associated with metastatic pathways: *platelet-derived growth factor-β* (*PDGFB*, a potent angiogenic, lymphangiogenic and transformative factor^16^); *lipocalin 2* (*LCN2* or *neutrophil gelatinase*, a protease associated with cell invasion and inflammatory response); and *lysyl oxidase-like 2* (*LOXL2*, a collagen hydroxylase associated with metastasis^17, 18, 19^) and were dependent on KDM4B in both oxygen tensions (Figure 3d). Regulation of *PDGFB*, *LCN2*, *LOXL2* and *LOX* (*lysyl oxidase*, another collagen hydroxylase associated with metastasis^19^) was validated in SKOV3ip.1 cells stably transduced with two small hairpin RNA (shRNA) constructs specifically targeting KDM4B (shK-1 and shK-2, Figure 3e, see Supplementary Figure S2 for specificity compared with other KDM4 family members). *LCN2* was decreased by ∼50% following KDM4B knockdown in normoxia and hypoxia, whereas hypoxic induction of *PDGFB*, *LOX* and *LOXL2* decreased by 25–30%. Knockdown of KDM4B in OVCAR8 cells decreased expression of PDGFB and IGFBP1 ∼50% in both normoxia and hypoxia (Supplementary Figure S2F). Increased expression of *LOX* in EOC patient tumor samples associates with poor progression-free survival,^20^ linking a putative KDM4B target gene to disease progression (Figure 3f). Combined, these data suggest KDM4B regulates distinct functions in different oxygen tensions, providing multiple avenues to affect EOC tumor growth.

### KDM4B binds and demethylates regulatory regions of target genes to promote expression

**Figure 4:**
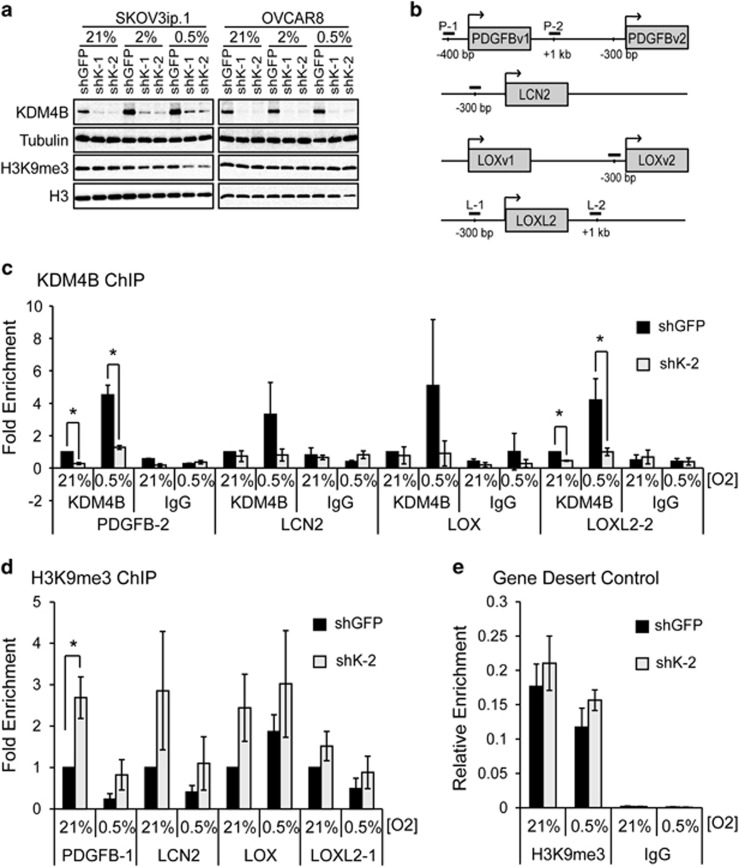
KDM4B binds regions proximal to target gene promoters and demethylates histone H3K9me3 to regulate expression. (**a**) Immunoblot measurement of KDM4B and H3K9me3 in SKOV3ip.1 and OVCAR8 cells expressing shRNA targeting KDM4B (shK-1 and shK-2) or GFP (control) in 21, 2 and 0.5% O_2_. Tubulin and histone H3 serve as loading controls. (**b**) Map of chromatin immunoprecipitation (ChIP) quantitative PCR (qPCR) primers sets used to measure KDM4B association and histone H3K9 methylation at or near target gene promoters. (**c**) ChIP assay for KDM4B near promoters of *PDGFB*, *LCN2*, *LOX* and *LOXL2* genes in SKOV3ip.1 cells expressing shRNA to KDM4B (shK-2, light gray bars) compared with shGFP control (black bars). Cells were treated with normoxia (21%) or hypoxia (0.5%) for 16 h. Data represent mean fold enrichment±s.e.m. of three independent experiments (*n*=3) measured in triplicate, normalized input and then to GFP control at 21% O_2_. (**d**) ChIP qPCR analysis of H3K9me3 on indicated promoter regions of the *PDGFB*, *LCN2*, *LOX and LOXL2* genes following shRNA to KDM4B (shK-2, light gray bars) compared with shGFP control (black bars). Data represent the mean of four independent experiments±s.e.m. (*n*=4) measured in triplicate, and normalized to shGFP control following subtraction of IgG signal and normalization to a gene desert control region as described in the Materials and methods. (**e**) H3K9me3 ChIP to a gene desert control region. Data represent the mean of four independent experiments±s.e.m. (*n*=4) measured in triplicate normalized to input. Significance relative to shGFP control was determined using paired Student's *t*-test (*P<0.05).

KDM4B primarily regulates gene expression by demethylating H3K9me3, enhancing transcription by removing a modification associated with repression.^21^ Stable loss of KDM4B did not result in any changes in bulk histone H3K9me3 in either SKOV3ip.1 or OVCAR8 cells under any oxygen tensions examined (Figure 4a). In order to determine whether KDM4B directly regulated putative target gene expression, SKOV3ip.1cells were exposed to 21 and 0.5% oxygen for 16 h, and chromatin immunoprecipitation was performed using antibodies against KDM4B and H3K9me3. In SKOV3ip.1-shGFP cells, KDM4B was localized to regions near the transcription start sites of *PDGFB*, *LCN2*, *LOX* and *LOXL2* (Figures 4b and c), with a 3–6-fold increase in association in 0.5% oxygen. Following knockdown, association of KDM4B to regulated promoters decreased to levels similar to an isotype-specific IgG control. Knockdown of KDM4B increased enrichment of H3K9me3 at the promoters of *PDGFB*, *LCN2*, *LOX* and *LOXL2* in 21% oxygen (Figure 4d). In 0.5% oxygen, knockdown of KDM4B elevated H3K9me3 compared with the control. However, the overall magnitude of association at *PDGFB*, *LCN2* and *LOX* decreased in hypoxia, making this difference less significant. The enrichment of H3K9me3 on a control ‘gene desert' region was not significantly affected by loss of KDM4B or by hypoxia (Figure 4e). These results demonstrate that KDM4B associates with regulatory regions of target genes, and demethylates histones residing near transcription start sites to regulate gene expression.

### KDM4B regulates cell invasion, migration and spheroid formation *in vitro*

**Figure 5:**
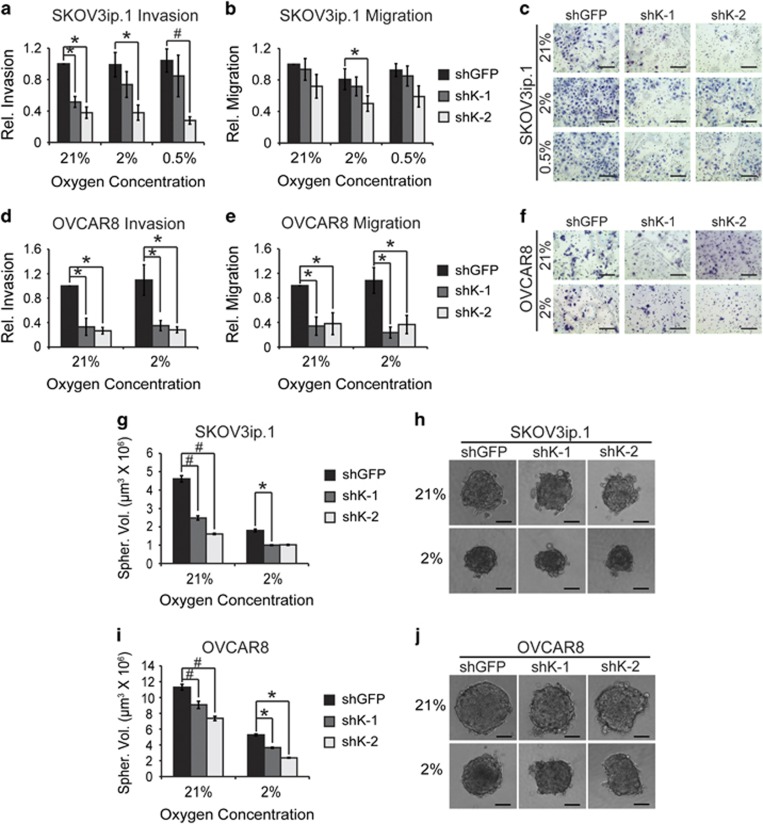
KDM4B supports ovarian cancer cell invasion, migration and 3D spheroid formation. (**a**) SKOV3ip.1 Matrigel Invasion Assay. 5 × 10^4^ SKOV3ip.1 cells expressing the indicated shRNA were seeded to Boyden chambers coated with Matrigel and cultured for 24 h in the indicated oxygen tensions. Cells were trypsinized and quantified as described in the Materials and methods. (**b**) Migration assay. 5 × 10^4^ SKOV3ip.1 cells were seeded to empty Boyden chambers, cultured and quantified as in (**a**). Data in (**a**) and (**b**) represent mean±s.e.m. for five independent experiments (*n*=3). (**c**) Representative image of SKOV3ip.1 invasion assay. Filters treated in parallel to those in (**a**) were fixed and stained, as described in the Materials and methods. × 100 magnification, scale bar, 200 μm. (**d**) OVCAR8 invasion assay performed as in (**a**). (**e**) Migration assay for OVCAR 8 cells, treated as in (**b**). Data in (**d**) and (**e**) represent the mean±s.e.m. for four independent experiments, *n*=3. (**f**) Representative images of OVCAR8 Matrigel invasion assay. × 100 magnification, scale bar, 200 μm. (**g**) SKOV3ip.1 spheroid formation assay. SKOV3ip.1 cells transduced with the indicated shRNA were cultured in ULA dishes in 21% and 2% O_2_ as described in the Materials and methods. After 4 days, spheroid volume was calculated. Data represent mean volume±s.e.m. *n*=16. (**h**) Representative images (median volume) of SKOV3ip.1 spheroids. × 100 magnification, scale bar, 100 μm. (**i**) OVCAR8 spheroid formation assay, conducted as described for (**g**). (**j**) Representative images (median volume) of OVCAR8 spheroids. × 100 magnification, scale bar, 100 μm. For (**a**, **b**, **d**, **e**, **g**, **i**), significance was calculated relative to shGFP control at each oxygen condition, using Mann–Whitney *U*-tests (*P<0.05; ^#^*P*<0.005).

In order to determine whether KDM4B mediatesmetastatic phenotypes, SKOV3ip.1 and OVCAR8 cells were seeded to Boyden chamber migration and invasion assays (Figures 5a–f). Following KDM4B knockdown, invasion through Matrigel was significantly reduced by ∼60% decrease in both cells lines (Figures 5a and c–e). Migration through uncoated inserts was decreased by ∼25–30% in SKOV3ip.1 cells (Figure 5b). In OVCAR8 cells, migration through uncoated chambers was severely attenuated (>80%, Figure 5e), indicating that although KDM4B was universally important for regulating invasive behavior in both cell lines, its effects on invasion and migration may be mediated through distinct but overlapping mechanisms. Stable knockdown of KDM4B had little effect on cellular doubling time in SKOV3ip.1 and OVCAR8 cells (Supplementary Figures S3A and B), nor were there significant differences between proliferation controls used for Boyden chamber assays (Supplementary Figures S3C and D).

Another characteristic of EOC is the tendency to form clusters of cells suspended in ascites that seed the peritoneal compartment.^4^ Using *in vitro* spheroid formation assays,^22^ loss of KDM4B significantly reduced the volume of spheroids by 50% in SKOV3ip.1 cells, with similar results in OVCAR8 cells (Figures 5g and i). In addition, the control spheroids were more rounded and smoother in appearance, indicating more robust cell-to-cell contact (Figures 5h and j). Combined, these results indicate that KDM4B may influence ovarian cancer progression by promoting formation of ascites spheroids in the peritoneal cavity and subsequent invasion of these clusters to peritoneal tissues.

### KDM4B regulates seeding and growth of peritoneal tumors

**Figure 6:**
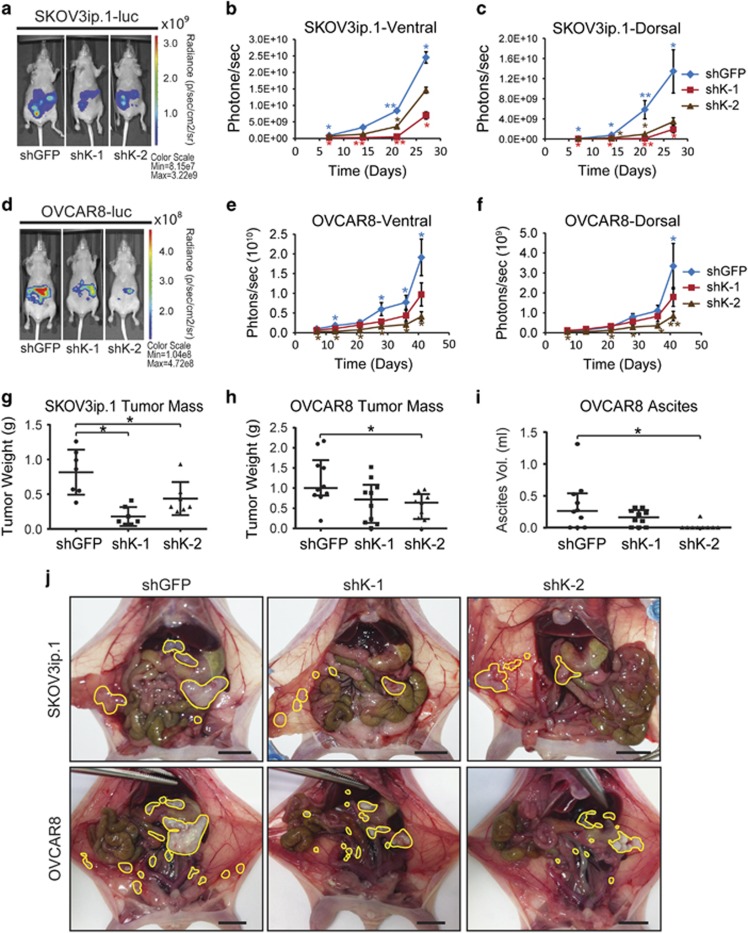
KDM4B regulates peritoneal seeding and growth of ovarian cancer cells. (**a**) Representative ventral bioluminescent images of nude mice injected with SKOV3ip.1-luc-neo cells transduced with indicated shRNA constructs. Image represents the median tumor load for each group, 28 days after injection. (**b**) Longitudinal ventral bioluminescence measurement for all mice injected with SKOV3ip.1-luc-neo transduced with indicated shRNA. Data represents mean total radiance (photons/sec) for each group±s.e.m. (*n*=9 or 10 mice per group). Blue diamonds, shGFP; red squares, shK-1; and brown triangles, shK-2. (**c**) Dorsal bioluminescence for SKOV3ip.1 study, analyzed as in (**b**). (**d**) Representative images of mice injected with OVCAR8-luc-neo cells transduced with the indicated shRNA constructs. Image was captured at week 6 of study. (**e**) Ventral bioluminescence measurement for all mice injected with OVCAR8-luc-neo transduced with indicated shRNA constructs, analyzed as in (**b**). (**f**) Dorsal bioluminescence for OVCAR8 study, analyzed as in (**b**). Statistics for (**b**), (**c**), (**e**) and (**f**) were calculated with Kruskal–Wallis and Mann–Whitney *U*-tests (*P<0.05; **P<0.005). Blue asterisks, difference among all three groups; red asterisks, difference between shK-1 and shGFP; brown asterisks, difference between shK-2 and shGFP. (**g**) Box-and-whisker dot plot of total tumor weight for SKOV3ip.1 xenografts (*n*=7 per group). (**h**) Dot plot of tumor weight for OVCAR8 xenografts (*n*=9 or 10). (**i**) Dot plot of ascites fluid volume collected from OVCAR8 experiment (*n*=9 or 10 mice per group). For (**g**–**i**), statistical significance was determined using Mann–Whitney *U*-test relative shGFP control (*P<0.05). (**j**) Representative end point necropsy demonstrating differential tumor size and distribution in mice injected with SKOV3ip.1 or OVCAR8 cells. Scale bar, 1 cm. Visible tumors are circled in yellow. Note the large cluster of fused omental tumors in the shGFP controls as compared with the knockdown mice.

**Figure 7:**
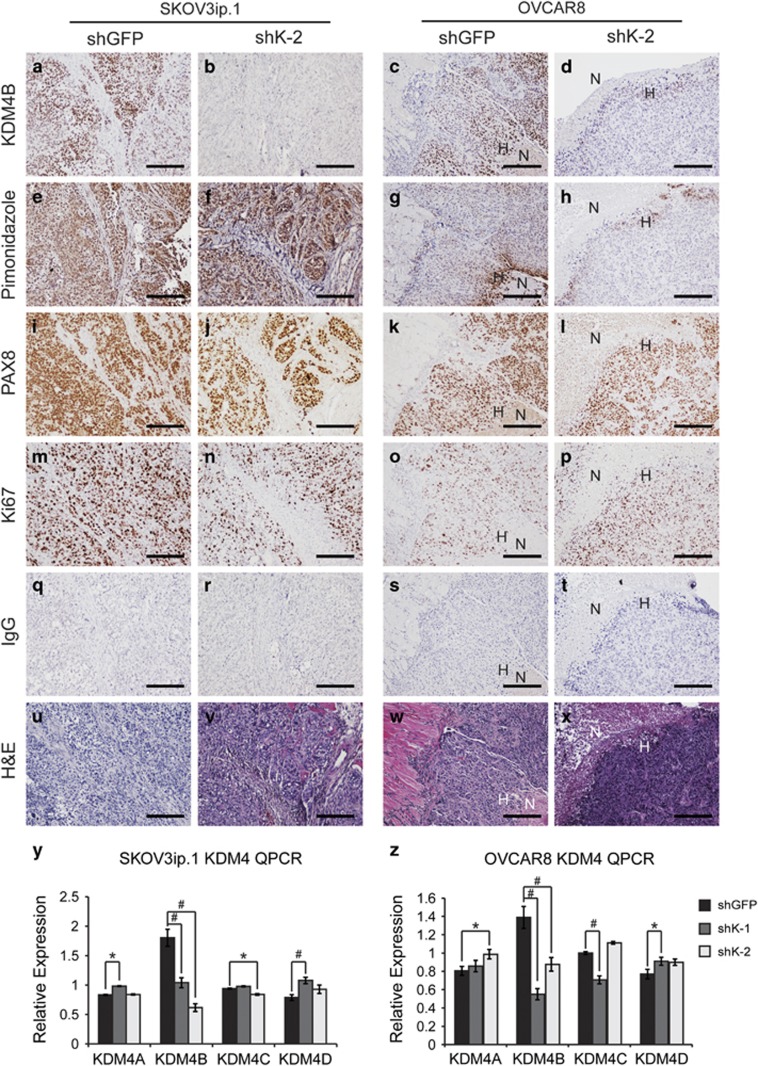
KDM4B is expressed in hypoxic regions of tumor xenografts. (**a–d**) KDM4B. (**e–h**) Pimonidazole. (**i–l**) PAX8. (**m–p**) Ki67. (**q–t**) IgG. (**u–x**) Hematoxylin and eosin (H&E). Scale bar, 100 μm. H, hypoxia; N, necrosis. (**a**, **e**, **i**, **m**, **q**) And (**u**) correspond to SKOV3ip.1 shGFP control tumor sections. (**b**, **f**, **j**, **n**, **r**) And (**v**) correspond to SKOV3ip.1 shK-2 KDM4B knockdown tumor sections. (**c**, **g**, **k**, **o**, **s**) And (**w**) correspond to OVCAR8 shGFP control tumors. (**d**, **h**, **l**, **p**, **t**) And (**x**) correspond to OVCAR8 shK-2 tumor sections. All immunohistochemical (IHC) sections were counterstained with hematoxylin to visualize cell nuclei. Images were captured at × 00X magnification. Scale bar, 200 μm. (**y**) Quantitative real-time PCR (qRT–PCR) measurement of KDM4 subfamily members in SKOV3ip.1 xenograft tumors, as described in the Materials and methods (shGFP, *n*=7; shK-1, *n*=7; shK-2, *n*=7). (**z**) The qRT–PCR measurement of KDM4 subfamily members in OVCAR8 xenograft tumors (shGFP, *n*=10; shK-1, *n*=9; shK-2, *n*=8). Data represent average fold change relative to shGFP control, after normalization to homo sapiens *GAPDH*. Significance was calculated by Mann–Whitney *U*-test relative to shGFP control (*P<0.05; #*P*<0.005). Note colocalization of KDM4B with pimonidazole staining near the necrotic core of the OVCAR8 shGFP biopsy (**c**, **g**).

The *in vivo* role of KDM4B in EOC tumor progression was determined using intraperitoneal tumor xenografts. Loss of KDM4B significantly inhibited peritoneal seeding and tumor growth for both SKOV3ip.1 and OVCAR8 cells, as measured by bioluminescence (Figure 6). This effect was significant for both constructs in SKOV3ip.1 cells (Figures 6a–c), whereas shK-2 showed the most significant effect in OVCAR8 cells, with a trend of reduced growth for shK-1 (Figures 6d–f). Total end point tumor weight was decreased by at least 50% in SKOV3ip.1 cells and OVCAR8 cells (Figures 6g and h). OVCAR8 cells expressing shRNA to KDM4B developed less ascites fluid compared with controls, consistent with the effect on tumor seeding and growth. (Figure 6i). Terminal necropsy confirmed a general decrease in the size of metastatic nodules in the omentum, viscera and peritoneal wall (Figure 6j). Immunohistochemical detection of KMD4B in xenograft tumors confirmed the stability of knockdown throughout the study (Figures 7a–d). KDM4B was expressed in hypoxic regions of tumor epithelium as determined by pimonidazole and PAX8 staining (Figures 7e–h and 7i–l, respectively). There was no apparent difference in cell proliferation (Ki-67 staining, Figures 7m–p), consistent with *in vitro* experiments (Supplementary Figure S3). The quantitative PCR measurement of *KDM4A*, *KDM4B*, *KDM4C* and *KDM4D* RNA in xenograft tumors (Figures 7y and z, respectively) confirmed stable knockdown of KDM4B while also identifying slight increases in expression of KDM4A and *KDM4D*. Combined, these studies demonstrate a role for hypoxic expression of KDM4B in the establishment and growth of peritoneal tumors.

## Discussion

**Figure 8:**
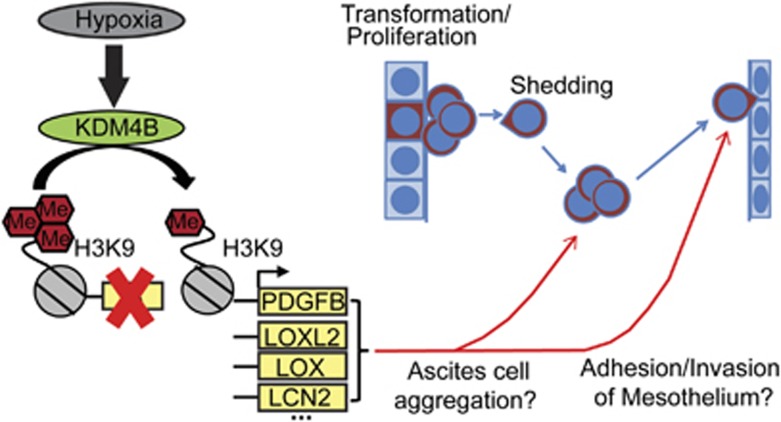
KDM4B regulates peritoneal seeding of ovarian cancer. Hypothesized mechanism of KDM4B signaling to promote ovarian cancer progression. Briefly, hypoxia induces the expression of KDM4B, catalyzing demethylation of histone H3K9me3 at target promoters and regulating expression of genes that facilitate the seeding of metastases in the viscera and peritoneal wall.

Developing more effective methods to attenuate peritoneal dissemination and tumor recurrence in ovarian cancer patients depends on improved understanding of the processes contributing to attachment-free growth of cancer cells in malignant ascites, invasion of cancer cells into the mesothelium and establishment of new tumors. Our data suggest that KDM4B contributes significantly to pathways that influence peritoneal seeding of tumors. As a hypoxia-inducible regulator of peritoneal seeding, KDM4B represents a novel mechanism used by EOC cells to promote progression of ovarian cancer (Figure 8).

Stable knockdown of KDM4B disrupted invasion and migration *in vitro*, consistent with diminished peritoneal tumor load in mouse xenografts. The most striking difference was in the size of omental tumors, suggesting that KDM4B may preferentially regulate metastasis to that tissue. Furthermore, the contribution of KDM4B to the formation and growth of tumor spheroids *in vitro* likely relates to *in vivo* function of KDM4B by facilitating peritoneal seeding.^4^ These phenotypes appear to be distinct from the actions of KDM4A in EOC and other cancers, where it promotes copy number gain and drug resistance.^23, 24^

A key question rising from our study is with regard to the utility of directly targeting KDM4B to improve EOC patient outcome. Although a systematic comparison has not been conducted, KDM4B appears to regulate pathways distinct to each cancer type studied. In hormone-dependent breast and prostate cancers, KDM4B is both a target and facilitator of hormone-dependent transcription.^25, 26^ In colon and gastric cancers, KDM4B regulates β-catenin-dependent gene expression to promote attachment-free growth and metastatic behavior.^27, 28^ In EOC, although KDM4B regulates distinct sets of genes in different oxygen tensions, invasive and migratory behaviors are the most robust phenotypes (Figures 3, 4, 5, 6). *PDGFB*, *LCN2*, *LOX* and *LOXL2* depend on KDM4B for expression, corresponding to changes in histone methylation on their respective promoters when KDM4B was knocked down. Considering the large number of genes dependent on KDM4B in SKOV3ip.1 cells, it is unclear which specific genes or pathways are primarily responsible for mediating metastasis, although *LCN2*, *LOX*, *LOXL2* and *PDGFB* make compelling candidates with proven abilities to influence either ovarian cancer growth or general metastasis.^17, 19, 29, 30^ Analysis of data sets curated by Gyorffy *et al.*^20^ demonstrated that elevated LOX expression in tumor samples correlated with reduced progression-free survival in EOC (Figure 3e). In an additional independent proof of concept, blocking PDGFB in SKOV3ip.1 cells with DNA aptamers suppressed growth of peritoneal tumor xenografts.^31^ These results support the potential roles of KDM4B target genes, identified in this study, in the pathogenesis of EOC. Combinatorial targeting of genes regulated by KDM4B may lead to improved methods to suppress peritoneal engraftment of EOC tumor cells, ultimately improving the prognosis of patients with elevated levels of KDM4B.

KDM4B regulates proliferative genes in transient, but not stable knockdown experiments (data not shown), raising the issue of compensatory mechanisms. Expression of KDM4D protein and RNA increased following knockdown of KDM4B *in vitro* and *in vivo* (Figures 7y and z and Supplementary Figure S2). Compensatory *Kdm4b* expression has been observed in the testes of *Kdm4d* knockout mice, possibly representing an inverse effect.^32^ Further supporting the overlapping roles of the KDM4 family members, simultaneous deletion of *Kdm-4a*, -*4b* and -*4c* was required to determine the role of Kdm4 family members in a mouse model of MLL-AF9-driven leukemia.^33^ These reports indicate that KDM4B does not act in isolation to regulate gene expression, highlighting the need for systematic in-depth studies of transcriptional mechanisms.

Multiple KDMs are regulated by HIFs,^11^ and may also influence the ability of KDM4B to regulate hypoxic gene expression in EOC. KDM4A is stabilized by hypoxia independently of HIF to facilitate gene amplification.^24^ KDM4 family members interact with H3K4me3 and H4K20me2 through tandem TUDOR domains, indicating that KDM4B may be recruited to specific regions of transcriptional activation or DNA damage.^34, 35^ The KDM4 family is generally inhibited by hypoxic conditions.^9, 36^ The hypoxia-inducible KDM3A, which can demethylate H3K9me2/1 in hypoxia,^9, 10^ may facilitate KDM4B function. Although in-depth investigations in ovarian cancer have not been conducted, KDM3A is induced >1.7-fold in hypoxic SKOV3ip.1 cells (Supplementary Table S6). Future experiments investigating regulation of EOC-promoting genes by HIFs, KDM3A and KDM4B will determine how or if KDM3A and KDM4B directly interact to regulate gene expression. The interaction of KDM4B with MLL2 in breast cancer cells suggests that KDM4B may reinforce its associations with promoters by recruiting the deposition of H3K4me3.^37^ Although it is not yet known if MLL2 contributes to EOC, future studies examining the interactions between KDM4B, HIF, other KDMs and lysine methyltransferases will clarify the transcriptional mechanisms mediated by KDM4B in hypoxia and EOC.

Our studies, like previous reports, imply that disrupting KDM4B function with a demethylase inhibitor may attenuate tumor growth. To advance KDM4B as a direct therapeutic target in EOC, it will be important to demonstrate the extent to which demethylase activity modulates the hypoxic response, invasive phenotype and *in vivo* growth properties of ovarian cancer cells. Given the aforementioned issues regarding possible cooperative and compensatory mechanisms, in-depth mechanistic studies would be best accomplished using KDM4B catalytic and targeting mutants introduced using CRISPR/Cas9 gene editing.

Identifying genes regulated by KDM4B may have greater therapeutic potential than identifying a demethylase inhibitor. Despite significant effort, the greatest success in the development of specific histone demethylase inhibitors has been restricted to inhibition of the KDM6 family.^38, 39, 40, 41, 42, 43^ A systematic analysis of KDM-inhibitor interactions reported significant crossreactivity for all inhibitors tested, highlighting the difficulty of developing a specific inhibitor.^44^ KDM4A and KDM4B regulate DNA repair and cell proliferation, possibly independently of histone demethylation.^35, 45^ Therefore, targeting the enzymatic activity of KDM4B may be less effective than targeting the pathways regulated by KDM4B in EOC.

Our findings are consistent with previous publications demonstrating that loss of KDM4B decreases tumorigenic behavior, while extending the field into a tumor type that has previously received little attention from the histone demethylase field. Collectively, this study demonstrates that the hypoxia-inducible histone demethylase KDM4B is robustly expressed in the majority of EOC tumors assayed and regulates cellular phenotypes associated with seeding of peritoneal tumors, the primary cause of EOC patient morbidity and mortality. To the best of our knowledge, this is the first functional demonstration that a Jumonji-domain histone demethylase contributes to EOC progression. Future studies investigating the specific transcriptional and functional mechanisms regulated by KDM4B and other histone demethylases may reveal novel therapeutic candidates to suppress reestablishment of peritoneal tumors in EOC patients.

## Materials and methods

### Cell lines and culture conditions

OVCAR cell lines were maintained in RPMI-1640 (Invitrogen, Carlsbad, CA, USA) supplemented with 10% heat-inactivated fetal bovine serum and 1% penicillin–streptomycin (Invitrogen). SKOV3ip.1 cells were from Dr Erinn Rankin (Stanford University, Stanford, CA, USA) with permission from Dr Gordon Mills (MD Anderson Cancer Center, Houston, TX, USA). OVCAR3, OVCAR4, OVCAR5, OVCAR8 and IGROV1 cells were from the NCI-Frederick Cancer DCTD Tumor/Cell line Repository (Frederick, MD, USA). OVCAR10, UPN275 and HIO80 cells were from Dr Andrew Godwin (KUMC, Kansas City, KS, USA). Upon receipt, cells were expanded in culture to establish early passage stocks. After transducing with lentivirus, cells were used for experiments within 1–2 months to minimize cell drift or contamination, and were periodically screened for mycoplasma. For transient knockdown of KDM4B, SKOV3ip.1 cells were transfected with a pool of small interfering RNAs targeting KDM4B or an irrelevant control (siK4B, Dharmacon siGenome Smartpool, GE Dharmacon, Lafayette, CO, USA) as previously described.^10^ For xenografts, cells were transduced with lentivirus to express firefly luciferase and selected in neomycin (pLenti PGK V5-LUC Neo (w623-2), from Eric Campeau (plasmid 21471, Addgene, Cambridge, MA, USA).^46^ For stable knockdown, cells were transduced with pLKO.1-shRNA constructs targeting KDM4B (TRCN0000018014 (shK-1) and TRCN0000018016 (shK-2, GE Dharmacon)) and selected in puromycin. For hypoxic treatment, cells were incubated for 16–24 h in Ruskinn InVivo300 glove-box hypoxic incubators (Baker, Sanford, ME, USA) set to desired oxygen tensions.

### TMA samples

Ovarian TMAs were constructed by the University of Kansas Cancer Center Biospecimen Repository Core Facility (Kansas City, KS, USA) using archival formalin-fixed, paraffin-embedded samples of primary ovarian carcinoma with matched metastatic tissue samples. Samples were identified from the pathology departmental archives of the University of Kansas Medical Center (KUMC) from 1998 to 2009. TMAs were composed of tumor samples from 48 patients with ovarian cancer: 14 patients with primary, recurrent and metastatic samples (including one patient with distant metastasis to the brain), 27 patients with primary and metastatic samples and 7 patients with primary and recurrent samples and included serous (30) mixed (14; 12 of which included a serous component), carcinosarcoma (1), clear cell (1), papillary carcinoma, not otherwise specified (NOS; 1) and adenocarcinoma, NOS (1) samples. For every sample, hematoxylin and eosin-stained slides were reviewed by a board-certified pathologist who selected tumor-rich areas. Using the semi-automated TMArrayer (Pathology Devices, Inc., Westminster, MD, USA) TMA paraffin blocks were assembled with triplicate 1.0 mm cores using the marked slide as a guide.

### Immunohistochemical analysis

Immunohistochemistry was performed using standard peroxidase/DAB methods and hematoxylin counterstain (see Supplementary Table S7 for antibodies and conditions). Representative images were captured using a Nikon (Melville, NY, USA) 80i microscope with a Photometrics CoolSNAP ES camera and NIS-Elements AR software (Nikon). Human TMA slides were scanned using an Aperio slide-scanning microscope (Leica, Buffalo Grove, IL, USA) at the Department of Pathology, University of Kansas Medical Center, and Department of Radiation Oncology, University of California San Francisco (San Francisco, CA, USA) (courtesy of Dr Denise Chan). Individual spot images were extracted using Aperio TMALab. Slides were blinded and independently evaluated by at least two pathologists (authors GT, TK and OWT). If >10% of tumor epithelium had nuclear staining of KDM4B, the section was classified as having high expression.^47^ For CA-IX staining, tumors were scored on a qualitative scale (0=no staining, 1=weak staining, 2=moderate staining, 3=strong staining). Scores for triplicate spots from each tumor sample were averaged for statistical analysis. SPSS (IBM, Armonk, NY, USA) was used to determine significance of CA-IX and KDM4B staining with bivariate correlation (Spearman's ρ).

### Quantitative real-time PCR

Quantitative real-time PCR was conducted as previously described using an ABI VIIA 7 Real-Time PCR System (Applied Biosystems, Thermo Fisher, Waltham, MA, USA) using 18S rRNA as an internal control.^10^ Primers (Supplementary Table S8) were designed using the Roche Universal Probe Library Design Tool (Roche Diagnostics Corporation, Indianapolis, IN, USA) (http://lifescience.roche.com/shop/products/universal-probelibrary-system-assay-design). Melt curve analysis confirmed formation of single amplicons of the expected size.

### Microarray analysis

RNA (100 ng) was profiled using GeneChip Human Exon 1.0 ST exonic transcript arrays (Affymetrix, Inc., Santa Clara, CA, USA). Normalization and differential gene expression analysis was performed in the Partek Genomic suite (v 6.5, Partek Inc., St Louis, MO, USA). All experiments were performed in triplicate. The exon arrays were RMA-background corrected, quantile-normalized and gene-level summarized using the Median Polish algorithm.^48^ The resulting log (base 2) transformed signal intensities were used in a two-way analysis of variance model to calculate differential expression. *P*-values were corrected for multiple-hypothesis testing by the Benjamini and Hochberg method.^49^

Genes displaying fold change of <−1.4 or lower following siK4B knockdown (*P*<0.05) compared with the siCon-transfected cells were considered to be regulated by KDM4B. GeneVenn (http://www.bioinformatics.org/gvenn/) identified overlapping expression between different oxygen tensions and knockdown constructs. Specific pathways influenced by KDM4B were identified using Ingenuity Pathway Analysis (Ingenuity Systems, Qiagen, Redwood City, CA, USA). Microarray data were deposited under accession number GSE66894 in the NCBI Gene Expression Omnibus.^50^

### Immunoblotting

Immunoblots were performed using standard procedures as previously described.^10^ See Supplementary Table S7 for antibody dilution information.

### Growth curves

Cells (50 000) were seeded in triplicate to 6 cm dishes, cultured in HeraCell 150 incubators (ThermoFisher Scientific, Inc., Waltham, MA, USA) set to desired oxygen tension, counted and reseeded every 3 days, as described by Welford *et al.*^51^

### Migration and Matrigel invasion assay

Invasion and migration assays were performed as described by Swenson-Fields *et al.*^52^ with minor modifications. Cells (50 000) suspended in RPMI+2% fetal bovine serum were seeded to Boyden chambers (8 μm pore size, BD Falcon, Corning, Tewksbury, MA, USA) suspended in RPMI+10% fetal bovine serum. For invasion assays, chambers were coated with 100 μl 1 mg/ml Matrigel (BD Biosciences, San Jose, CA, USA). After 24 h of incubation (see ‘Cell lines and culture conditions' section), cells were released by trypsinization and DNA content quantified using CyQUANT Cell Proliferation Assay Kit (Life Technologies, ThermoFisher) according to the manufacturer's protocol. Invasion and migration were normalized to seeding and monolayer proliferation controls. Representative membranes were fixed and stained with Siemens Diff Quik Stain Set (Siemens, Inc., Malvern, PA, USA) and imaged using a Leitz LABORLUX12 microscope equipped with a Leica EC3 camera.

### Spheroid formation assay

A total of 600 cells were seeded per well of 96-well ultra-low attachment plates (Corning Incorporated, Corning, NY, USA). After 4 days, spheroids were imaged by bright-field microscopy using a Leica DMI 4000 inverted microscope equipped with a Leica CCD camera (Courtesy of Dr Michael J Soares, KUMC). Volume (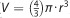) was calculated after measuring the diameter and radius of each spheroid using NIH Image J (National Institutes of Health, Bethesda, MD, USA).^22^ Differences in average spheroid volume were analyzed by two-tailed paired Student's *t*-test.

### Intraperitoneal tumor xenografts

Cells (1 000 000 SKOV3ip.1-luc-neo or 5 000 000 OVCAR8-luc-neo) were injected intraperitoneally into NCR-nu/nu athymic mice (Taconic, Hudson, NY, USA; 4–6-week-old females, 10 mice per group, randomly assigned for injection). Study numbers allow detection of an effect size of 1.6 relative to control with a power of 85% (*P*<0.05 using Student's *t*-test), assuming a tumor take rate of 80%. Tumor growth was monitored weekly (nonblinded) using the IVIS Spectrum *in vivo* imaging system (Perkin Elmer, Waltham, MA, USA) in the KUMC Center for Molecular Imaging until shGFP control mice achieved humane end points. Mice prematurely removed from the study were excluded from analysis. Mice were injected with 200–250 μl D-Luciferin (Gold Biotechnology, Inc., St Louis, MO, USA) 15 min before imaging. Pimonidazole (Hypoxyprobe, HPI, Burlington, MA, USA) was injected intraperitoneally 30 min before killing to label hypoxic cells. Portions of the largest tumors (primarily omental masses) were fixed in 4% paraformaldehyde and processed for immunohistochemistry. Frozen tumor samples were homogenized for RNA purification and analysis. Statistical significance was determined as described in the Statistics section.

### Chromatin immunoprecipitation assay

Chromatin immunoprecipitation was performed as described previously,^10^ with minor modifications. Sonicated DNA (8–50 μg) was incubated with 1–5 μg antibody (Supplementary Table S7). Enrichment to promoters and regulatory regions was measured using quantitative PCR calibrated to a dilution series of pooled input DNA and normalized to the respective input controls. For histone H3K9me3 analysis, input normalized signal was adjusted by subtracting IgG signal, with further normalization to gene desert control (C16D8). Fold changes in enrichment were calculated relative to shGFP control cells at 21% O_2_. Primers to promoters and control regions were designed using Primer Express software (Life Technologies, ThermoFisher) and validated for amplification efficiency and generation of a single amplicon (Supplementary Table S8).

### Statistics

Data represent the mean±s.e.m. or median±upper and lower quartiles for at least three independent experiments. Human tumor data and mouse xenografts were analyzed with nonparametric methods such as Kruskal–Wallis, Mann–Whitney *U*-test and Spearman's correlation to account for non-normal distributions of data. Two-tailed Student's *t*-tests or analysis of variance were used as appropriate for *in vitro* experiments. *P-*values of 0.05 were considered statistically significant.

### Study approvals

Animal studies were approved by the KUMC institutional animal care and use committee. Deidentified human patient samples and any corresponding clinical data were provided by the KU Cancer Center's Biospecimen Repository Core Facility (BRCF) with informed consent from patients. Collection and use of deidentified human patient samples for this study was approved by the KUMC Human Subjects Committee.
