# Supplementary material for: The histone demethylase KDM4B regulates peritoneal seeding of ovarian cancer
Source: Oncogene. 2016 Nov 21;36(18):2565–76. doi: 10.1038/onc.2016.412 (PMC5418103; doi:10.1038/onc.2016.412)
Supplement: Supplementary Figure Legends [file onc2016412x1.docx]

**Supplemental material for**

**The histone demethylase KDM4B regulates peritoneal seeding of ovarian cancer**

Cailin Wilson and Lei Qiu ^1,2 a^, Yan Hong^1^, Tejashree Karnik^2^, Guirguiss Tadros^2^, Brian Mau^2^, Tammy Ma^2^,Ying Mu^1^, Jacob New^3^, Raymond J. Louie^4^, Sumedha Gunewardena^5^, Andrew K. Godwin^2^, Ossama W. Tawfik^2^, Jeremy Chien^6^, Katherine F. Roby^7^, Adam J. Krieg^1,2,8^ *

^1^Department of Obstetrics and Gynecology

University of Kansas Medical Center, Kansas City, KS 66160, USA

^2^Department of Pathology and Laboratory Medicine

University of Kansas Medical Center, Kansas City, KS 66160, USA

^3^Medical Scholars Program

University of Kansas Medical Center, Kansas City, KS 66160, USA

^4^Department of Radiation Oncology

University of California, San Francisco, San Francisco, CA 94115, USA

^5^Department of Molecular and Integrative Physiology

University of Kansas Medical Center, Kansas City, KS 66160, USA

^6^Department of Cancer Biology

University of Kansas Medical Center, Kansas City, KS 66160, USA

^7^Department of Anatomy and Cell Biology

University of Kansas Medical Center, Kansas City, KS, 66160

^8^Current Institution: Department of Obstetrics and Gynecology

Oregon Health and Science University, Portland, OR 97239

**Supplemental Tables and Figures.**

**Supplemental Table S1.** Summary table of KDM4B expression in EOC TMAs, includes data from normal ovary, primary EOC tumors, and matched metastatic tumors. (Excel file)

**Supplemental Table S2.** Table of the average KDM4B and CA-IX immunohistochemistry scores for triplicate patient samples included in the TMA analysis for this study (Excel file).

**Supplemental Table S3.** Ingenuity Pathway Analysis of functional pathways regulated by KDM4B in normoxia and hypoxia (Excel file).

**Supplemental Table S4.** Microarray gene list (Excel file) – Genes down-regulated by siRNA to KDM4B in normoxic SKOV3ip.1 cells (P<0.05).

**Supplemental Table S5.** Microarray gene list (Excel file) – Genes down-regulated by siRNA to KDM4B in hypoxic SKOV3ip.1 cells (P<0.05).

**Supplemental Table S6.** Microarray gene list (Excel file) – Genes induced in hypoxia compared to normoxia in SKOV3ip.1 cells transfected with control siRNA (P<0.05).

**Supplemental Table S7.** List of antibodies used in the study (Excel file).

**Supplemental Table S8.** List of primers used for QRT-PCR expression analysis and ChIP-QPCR analysis (Excel file).

**Supplemental Figure 1.** Modified Histone Peptide Array.

**Supplemental Figure 2.** KDM4B Regulates Expression of PDGFB and IGFBP1 in OVCAR8 expressing shRNA to KDM4B.

**Supplemental Figure 3.** Suppression of KDM4B Does Not Regulate Proliferation In SKOV3ip.1 and OVACR8 cells.

**Supplemental Figure Legends**

**Supplemental Figure S1.** **Modified Histone Peptide Array.** **A,** Specificity Plot demonstrating that the H3K9me3 antibody (Abcam ab8898) used for immunoblotting and ChIP was more specific for H3K9me3 than other modifications, including H3K27me3. **B,** Reactivity Plot for Abcam ab8898, demonstrating preferential reactivity with H3K9me3. Data was analyzed and figures generated with Array Analyze Software (Active Motif).

**Supplemental Figure S2.** **KDM4B Regulates** **Expression of PDGFB and IGFBP1 in OVCAR8 expressing shRNA to KDM4B.**. **A.** Immunoblotting of KDM4B, H3K9me3, H3K9me2, H3K9me1, H3K36me3, H3K36me2, and H3K36me1 expression in OVCAR8 cells. Histone H3 serve as control for protein loading. **B**. Quantitative RT-PCR measurement of KDM4 subfamily members in SKOV3ip.1 cells. **C**. Immunoblot of KDM4 subfamily members in SKOV3ip.1 cells. Tubulin serves as a loading control. **D.** Quantitative RT-PCR measurement of KDM4 subfamily members in OVCAR8 cells. Data represent mean ± S.E.M., normalized to 18S rRNA and shGFP control at 21% O2. Results were averaged from three independent experiments, measured in triplicate. *, P<0.05, determined by two-tails paired Student’s t-test. **E.** Immunoblot of KDM4 subfamily members in OVCAR8 cells. Tubulin serves as a loading control. **F.** Quantitative RT-PCR measurement of *PDGFB* and *IGFBP1* in OVCAR8 cells expressing shRNA to KDM4B (shK-1, dark grey and shK-2, light grey) in 21%, and 0.5% oxygen. QPCR data in panels B, D, and F represent the mean ± S.E.M., normalized to 18S rRNA and shGFP control at 21% O2. Results were averaged from three independent experiments, measured in triplicate. *, P<0.05, determined by two-tailed paired Student’s t-test.

**Supplemental Figure S3.** **Suppression of** **KDM4B Does Not Regulate Proliferation In SKOV3ip.1 and OVACR8 cells.** **A.** SKOV3ip.1 cells transduced with shRNA to KDMB (shK-1 and shK-2) were seeded for growth curve experiments as described in Methods, and cultured in 21% O_2_ and 2% O_2_. Data represent four independent experiments conducted in triplicate. **B**. OVCAR8 cells transduced with shRNA to KDMB (shK-1 and shK-2) were seeded for growth curve experiments as described in Supplemental Methods, and cultured in 21% O2 and 1% O2. Data represent three independent experiments, conducted in triplicate. Data in panels **A** and **B** represent mean ± S.E.M. #, P<0.05, comparing shK-1 to shGFP; *, P<0.05, comparing shK-2 to shGFP; determined by two-tailed paired Student’s t-test. **C-D,** Proliferation controls for Boyden Chamber Migration and Matrigel Invasion Assay. 25 000 cells were seeded as monolayers in parallel with cells seeded for Boyden chamber assays. After 24 hours, cells were trypsinized, frozen, and analyzed as described in Methods. Data represent mean ± S.E.M. Results were averaged from five (SKOV3ip.1, **C**) or four (OVCAR8, **D**) independent experiments conducted in triplicate. * = P<0.05, determined by two-tailed paired Student’s t-test.
