# Supplementary figures and images for: The histone demethylase KDM4B regulates peritoneal seeding of ovarian cancer
Source: Oncogene. 2016 Nov 21;36(18):2565–76. doi: 10.1038/onc.2016.412 (PMC5418103; doi:10.1038/onc.2016.412)

A

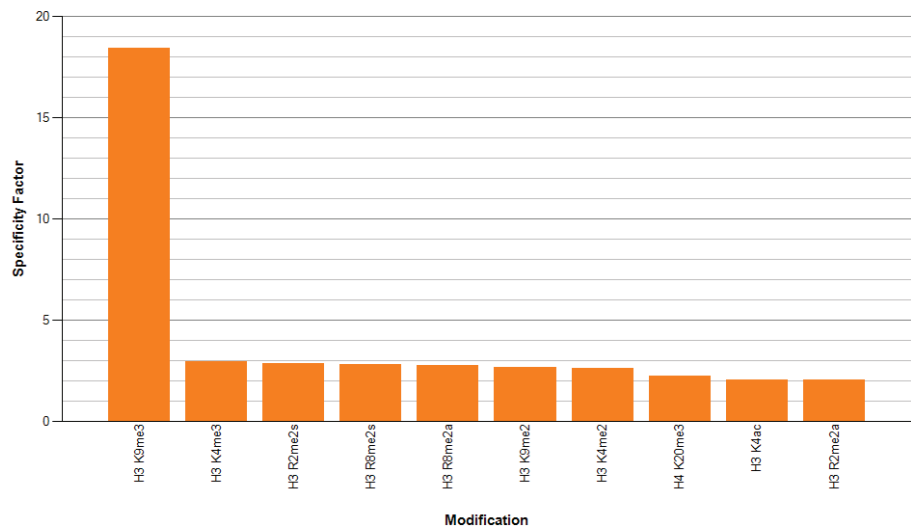

B

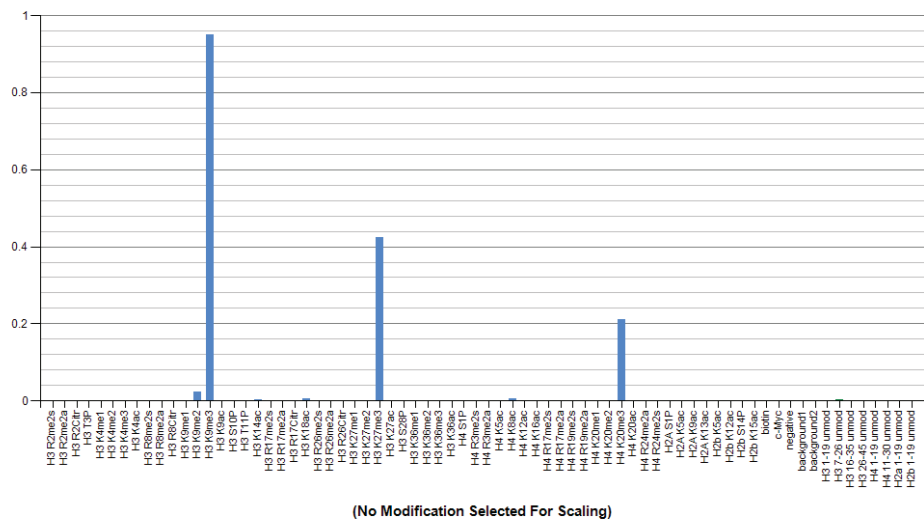

Supplement: Supplementary Figure S1 [file onc2016412x2.pdf]

A

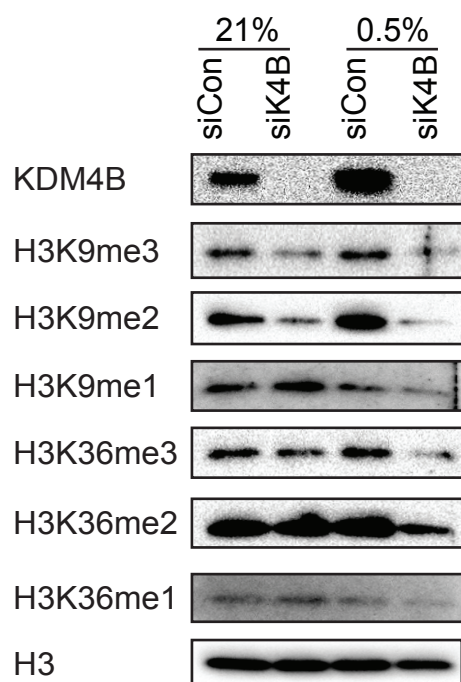

B

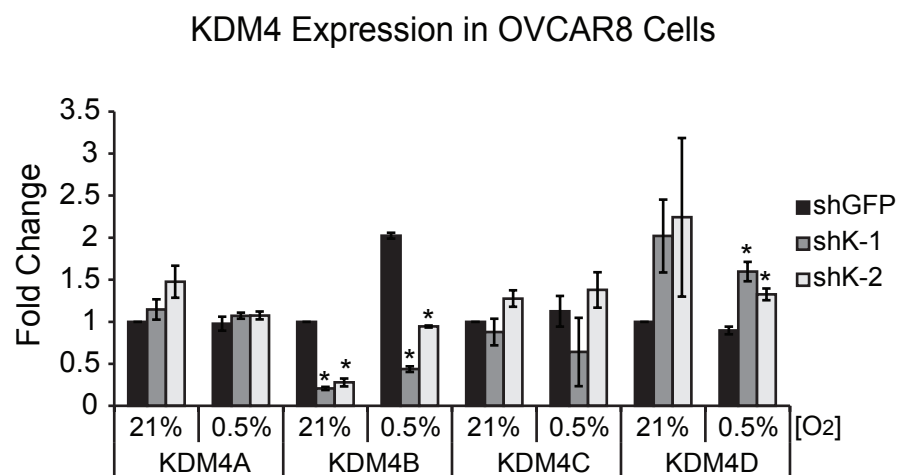

C

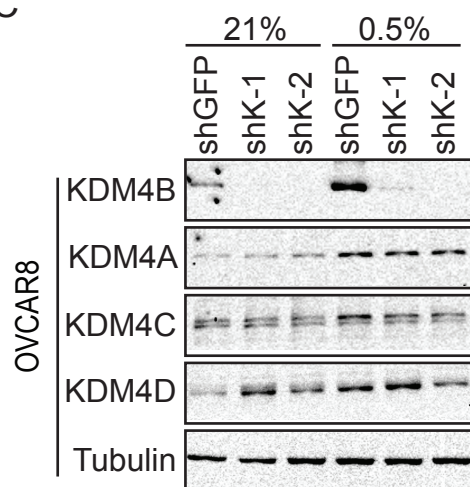

D

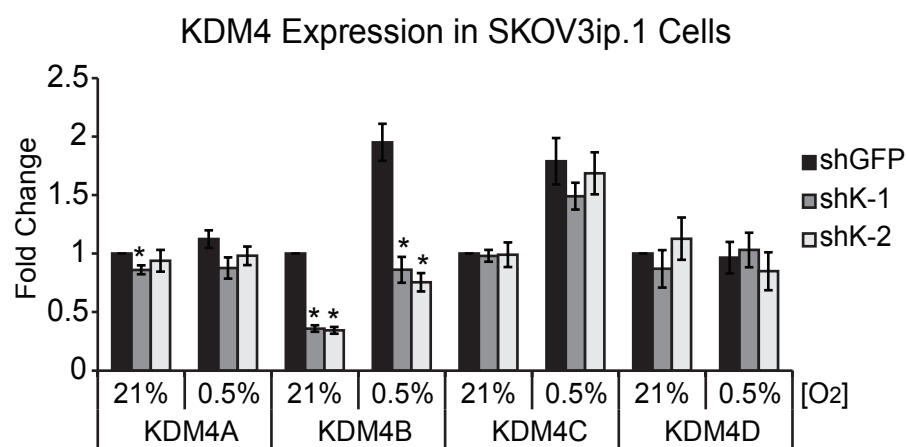

E

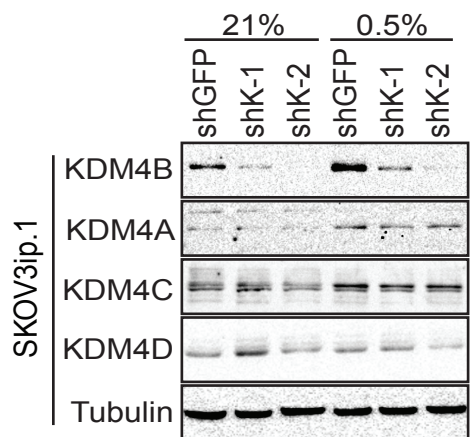

F

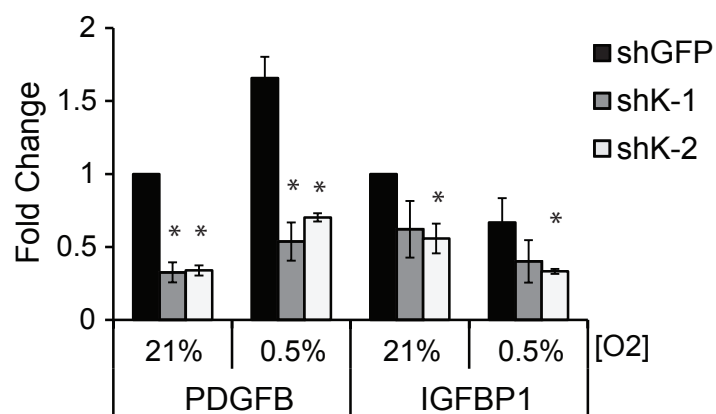

Supplement: Supplementary Figure S2 [file onc2016412x3.pdf]

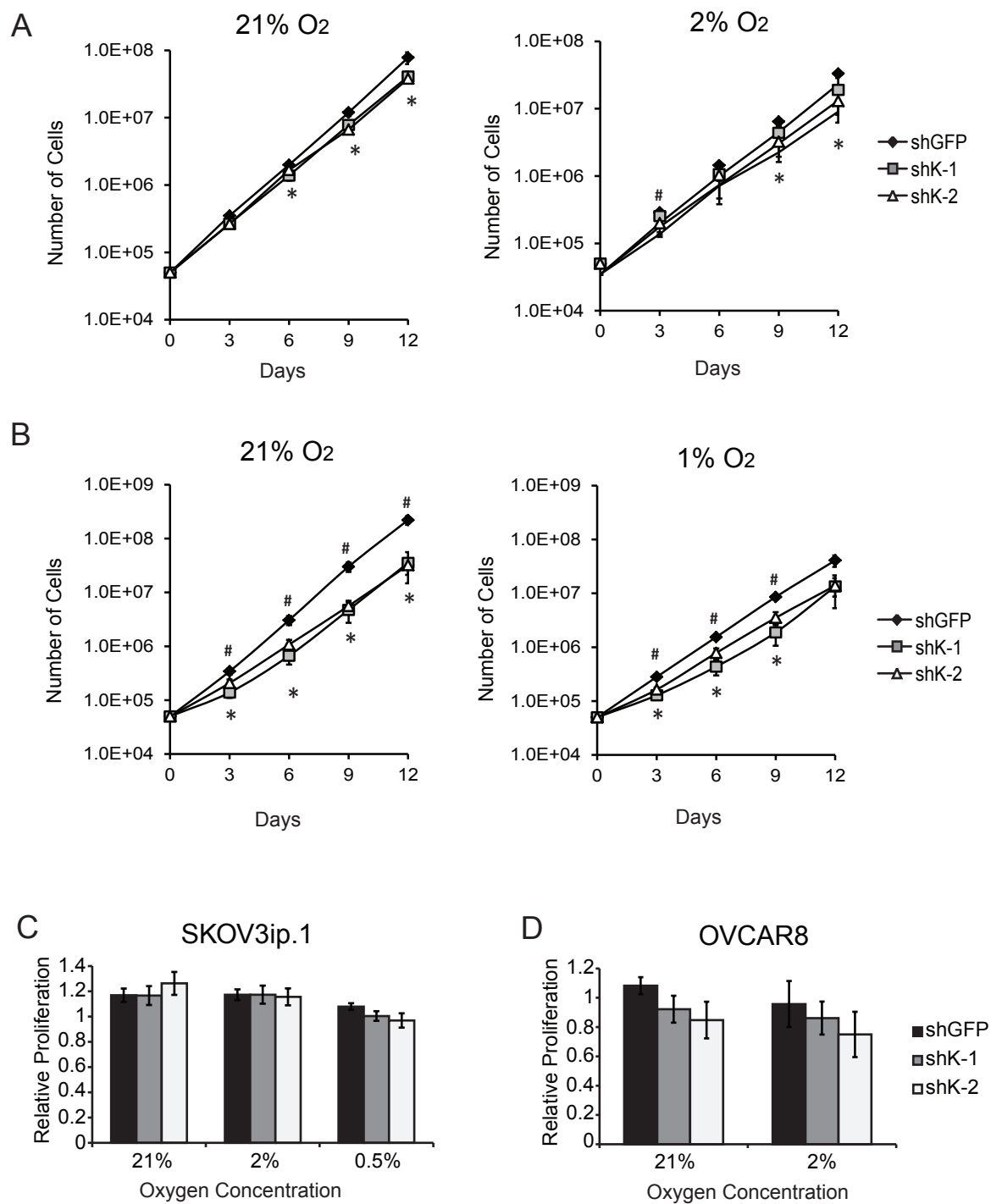

Supplement: Supplementary Figure S3 [file onc2016412x4.pdf]
